# Longitudinal long term follow up investigation on the carcinogenic impact of polyhexamethylene guanidine phosphate in rat models

**DOI:** 10.1038/s41598-024-57605-x

**Published:** 2024-03-26

**Authors:** Sang Hoon Jeong, Hong Lee, Yoon Jeong Nam, Ja Young Kang, Hyejin Lee, Jin Young Choi, Yu-Seon Lee, Jaeyoung Kim, Yoon Hee Park, Su A. Park, Hangseok Choi, Eun-Kee Park, Yong-Wook Baek, Jungyun Lim, Suejin Kim, Cherry Kim, Ju-Han Lee

**Affiliations:** 1 Medical Science Research Center, Ansan Hospital, Korea University College of Medicine, 123, Jeokgeum-ro, Danwon-gu, Ansan-si, Gyeonggi 15355 South Korea; 2 Medical Science Research Center, Korea University College of Medicine, 73, Goryeodae-ro, Seongbuk-gu, Seoul, 02841 South Korea; 3 Department of Medical Humanities and Social Medicine, College of Medicine, Kosin University, Busan, 49267 South Korea; 4 Humidifier disinfectant Health Center, National Institute of Environmental Research, Incheon, 22689 South Korea; 5 Environmental Health Research Division, National Institute of Environmental Research, Incheon, 22689 South Korea; 6 Department of Radiology, Ansan Hospital, Korea University College of Medicine, 123, Jeokgeum-ro, Danwon-gu, Ansan-si, Gyeonggi 15355 South Korea; 7 Department of Pathology, Ansan Hospital, Korea University College of Medicine, 123, Jeokgeum-ro, Danwon-gu, Ansan-si, Gyeonggi 15355 South Korea

**Keywords:** Rat model, Polyhexamethylene guanidine phosphate, Chest CT, Lung cancer, Lung fibrosis, Cancer epidemiology, Cancer imaging, Lung cancer, Cancer, Environmental sciences, Health care, Pathogenesis

## Abstract

Polyhexamethylene guanidine phosphate (PHMG-p) is a major component in humidifier disinfectants, which cause life-threatening lung injuries. However, to our knowledge, no published studies have investigated associations between PHMG-p dose and lung damage severity with long-term follow-up. Therefore, we evaluated longitudinal dose-dependent changes in lung injuries using repeated chest computed tomography (CT). Rats were exposed to low (0.2 mg/kg, n = 10), intermediate (1.0 mg/kg, n = 10), and high (5.0 mg/kg, n = 10) doses of PHMG-p. All rats underwent repeated CT scans after 10 and 40 weeks following the first exposure. All CT images were quantitatively analyzed using commercial software. Inflammation/fibrosis and tumor counts underwent histopathological evaluation. In both radiological and histopathologic results, the lung damage severity increased as the PHMG-p dose increased. Moreover, the number, size, and malignancy of the lung tumors increased as the dose increased. Bronchiolar–alveolar hyperplasia developed in all groups. During follow-up, there was intergroup variation in bronchiolar–alveolar hyperplasia progression, although bronchiolar–alveolar adenomas or carcinomas usually increase in size over time. Thirty-three carcinomas were detected in the high-dose group in two rats. Overall, lung damage from PHMG-p and the number and malignancy of lung tumors were shown to be dose-dependent in a rat model using repeated chest CT scans during a long-term follow-up.

## Introduction

Polyhexamethylene guanidine phosphate (PHMG-p) is a major component of humidifier disinfectants (HDs), which are usually mixed with the water that is dispersed in vaporized form from humidifiers in South Korea to prevent microbial contamination. However, following the outbreak of HD-related lung injuries among many HD users in 2011, many epidemiological and clinical studies have reported that PHMG-p inhalation can cause severe lung injuries^1–4^.

Moreover, it has been well demonstrated that PHMG-p-induced lung injuries and diseases, including fibrosis, inflammation, and tumors; PHMG-p has also been shown to cause genetic alterations in animal studies^5–14^. In a short-term animal study, despite a single exposure of PHMG-p, at least one lung lesion appeared in all rats at each analysis week (1, 2, 4, 6, and 8 weeks after PHMG-p instillation). These lesions significantly changed over 8 weeks, as evidenced by chest computed tomography (CT) and histopathological examinations^5^. A long-term animal study showed increased severity of lung fibrosis over time. Additionally, persistent fibrosis, lung carcinomas, and many bronchiolar–alveolar hyperplasia/adenomas developed 1 year after a single exposure to PHMG-p^6^. However, as those studies used the same dose of PHMG-p (0.9 mg/kg), no evidence exists regarding the degree of lung injuries according to the dose of PHMG-p exposure.

Meanwhile, another toxic chemical, polyhexamethylene biguanide (PHMB), has a similar structure to PHMG-p. A previous study reported that PHMB exacerbated rat liver tumors^15^, and PHMB was classified as a second-class carcinogen by the European Chemicals Agency in 2011^16^. According to the structure–activity relationship, which is a principle common to all such molecules and assumes that molecules with similar structures possess similar activities, PHMG-p could also be carcinogenic. Additionally, although the recognized range of HD-related diseases has been expanded, there is a limitation in the level of evidence, with one epidemiological and clinical study commenting that lung cancer has not currently been recognized as being associated with exposure to HD^17^. Therefore, it is necessary to explore the carcinogenicity of PHMG-p using a long-term follow-up study conducted in a rat model.

Previous studies have demonstrated the possibility of evaluating lung lesions, including lung fibrosis and tumors, using conventional chest CT (even without micro-CT capacity)^5,6,18^. One of the greatest advantages of a chest CT is that it can facilitate diagnoses without needing biopsies. For example, a chest CT can diagnose lung fibrosis or bronchiectasis without histopathologic confirmation^7^, and it is the imaging modality most used to diagnose lung cancer. Repeated chest CT scans can also demonstrate longitudinal changes in individual lung lesions over time, which can facilitate the monitoring of lesions associated with PHMG-p exposure, such as fibrosis and tumors.

This study aimed to evaluate carcinogenic effects and longitudinal changes in lung injuries according to PHMG-p dosage in a rat model using repeated conventional chest CT scans to conduct a long-term follow-up.

## Results

### Lung lesion changes on chest CT between 10 and 40 weeks after treatment

Table 1 shows the comparisons of quantitative CT analysis results between 10 and 40 weeks after the first intratracheal instillations in each group. In the normal saline group, the mean lesion volume percentage was lower after 40 weeks than after 10 weeks, although there was no significant difference (5.22 ± 2.06% vs. 3.21 ± 1.70%, P = 0.133). In the low-dose group, the mean lesion volume percentage did not change significantly (14.60 ± 5.50% vs. 14.30 ± 4.56%, P = 0.479). In the intermediate-dose group, the mean lesion volume percentage decreased after 40 weeks (27.69 ± 6.70% vs. 23.92 ± 3.05%, P = 0.508). However, the mean lesion volume percentage was increased in the high-dose group (29.44 ± 3.73% vs. 34.92 ± 11.99%, P = 0.438). Additionally, the mean whole-lung volume in the high-dose group was reduced after 40 weeks (16.00 ± 2.27 mL vs. 14.51 ± 4.50 mL, P = 0.503). However, no significant differences were noted.

**Table 1:** Comparison of quantitative analysis of CT results for 10 weeks vs. 40 weeks after the first intratracheal instillation.

Group	Mean lesion volume (mL)			Mean lesion volume percentage			Mean whole lung volume (mL)		
	After 10 weeks	After 40 weeks	P-value	After 10 weeks	After 40 weeks	P-value	After 10 weeks	After 40 weeks	P-value
Naïve type	0.37 ± 0.18	0.42 ± 0.20	0.379	2.60 ± 1.10	2.88 ± 1.18	0.437	14.11 ± 1.23	14.58 ± 2.35	0.354
Normal saline	0.71 ± 0.26	0.51 ± 0.31	0.291	5.22 ± 2.06	3.21 ± 1.70	0.133	13.77 ± 1.21	15.67 ± 1.62	0.003
Low-dose	2.18 ± 0.66	2.18 ± 0.63	0.723	14.60 ± 5.50	14.30 ± 4.56	0.479	15.17 ± 1.37	15.41 ± 1.41	0.746
Intermediate-dose	3.92 ± 0.62	3.59 ± 0.61	0.510	27.69 ± 6.70	23.92 ± 3.05	0.508	14.50 ± 1.90	15.04 ± 2.03	0.788
High-dose	4.70 ± 0.80	4.68 ± 0.86	0.677	29.44 ± 3.73	34.92 ± 11.99	0.438	16.00 ± 2.27	14.51 ± 4.50	0.503

**Figure 1:**
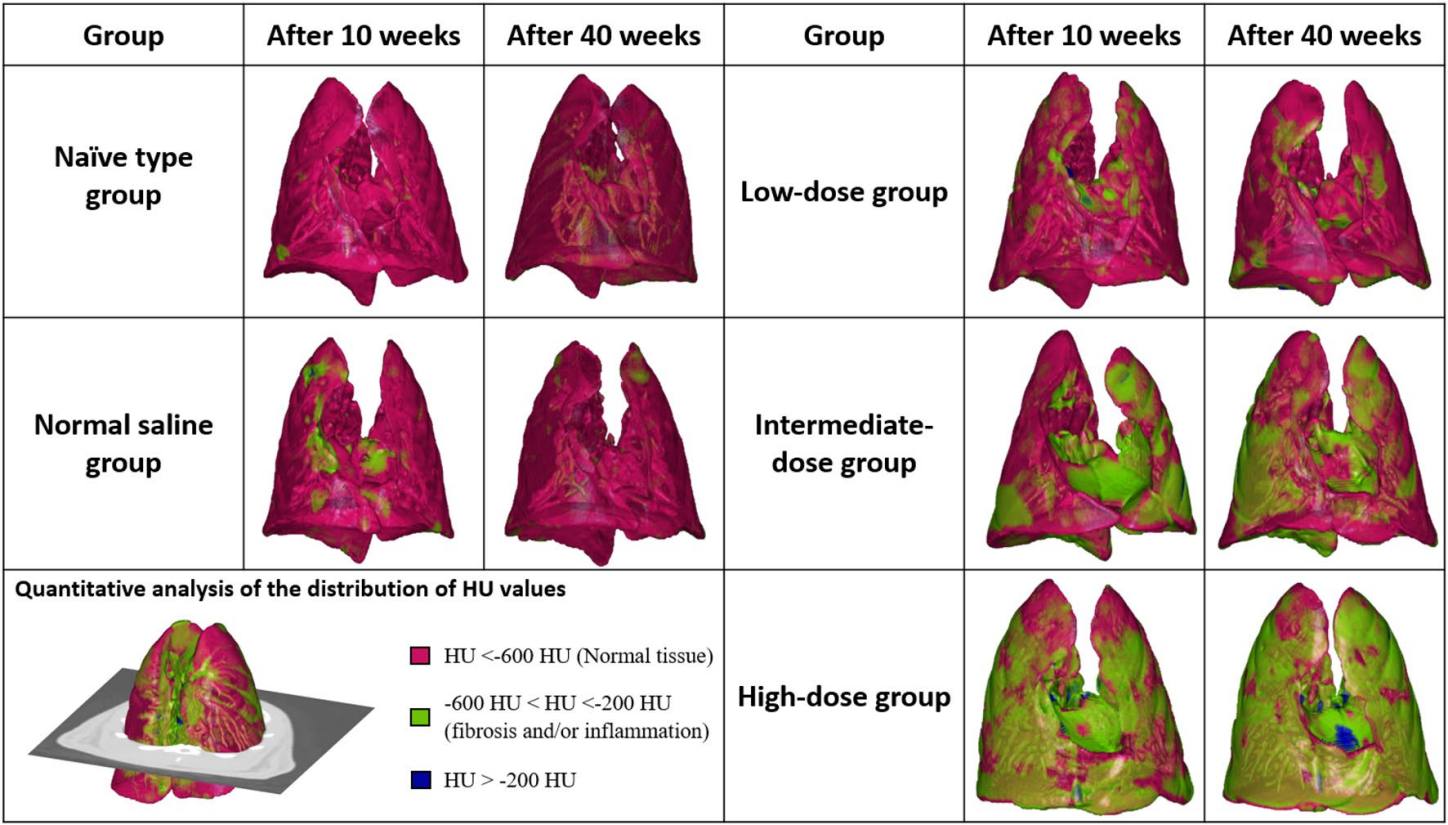
The schematic summary of the CT results. The quantitative analysis was performed using the distribution of the HU values of the pixels by generating normalized histograms.

Table 2, Fig. 1, and Supplementary Fig. 1 demonstrate the comparisons of the CT results between the groups, whereby the lung lesion volume increased significantly as the dose increased. The mean lesion volumes and mean lesion volume percentages significantly increased in the low-, intermediate-, and high-dose groups after 10 and 40 weeks compared with the naïve type and normal saline groups. Additionally, the high-dose group had a significantly higher mean lesion volume and mean lesion volume percentage than the low- and intermediate-dose groups after 10 and 40 weeks.

**Table 2:** The comparison of CT results between groups at 10 weeks vs. 40 weeks after the first intratracheal installation.

Group	Mean lesion volume (mL)	P-value	Mean lesion volume percentage	P-value	Mean whole lung volume (mL)	P-value
After 10 weeks						
Naïve type	0.37 ± 0.18	< 0.001	2.60 ± 1.10	< 0.001	14.11 ± 1.23	0.067
Normal saline	0.71 ± 0.26*		5.22 ± 2.06*		13.77 ± 1.21	
Low-dose	2.18 ± 0.66*^†§^		14.60 ± 5.50*^†§^		15.17 ± 1.37	
Intermediate-dose	3.92 ± 0.62*^†‡^		27.69 ± 6.70*^†‡^		14.50 ± 1.90	
High-dose	4.70 ± 0.80*^†‡§^		29.44 ± 3.73*^†‡§^		16.00 ± 2.27	
After 40 weeks						
Naïve type	0.42 ± 0.20	< 0.001	2.88 ± 1.18	< 0.001	14.58 ± 2.35	0.851
Normal saline	0.51 ± 0.31		3.21 ± 1.70		15.67 ± 1.62	
Low-dose	2.18 ± 0.63*^†^		14.30 ± 4.56*^†^		15.41 ± 1.41	
Intermediate-dose	3.59 ± 0.61*^†^		23.92 ± 3.05*^†^		15.04 ± 2.03	
High-dose	4.68 ± 0.86*^†‡^		34.92 ± 11.99*^†‡§^		14.51 ± 4.50	

*P < 0.05 (vs. naïve type group), ^†^P < 0.05 (vs. normal saline group), ^‡^P < 0.05 (vs. low-dose group), ^§^P < 0.05 (vs. intermediate-dose group).

### Histopathologic changes between 10 and 40 weeks after the first treatment

**Figure 2:**
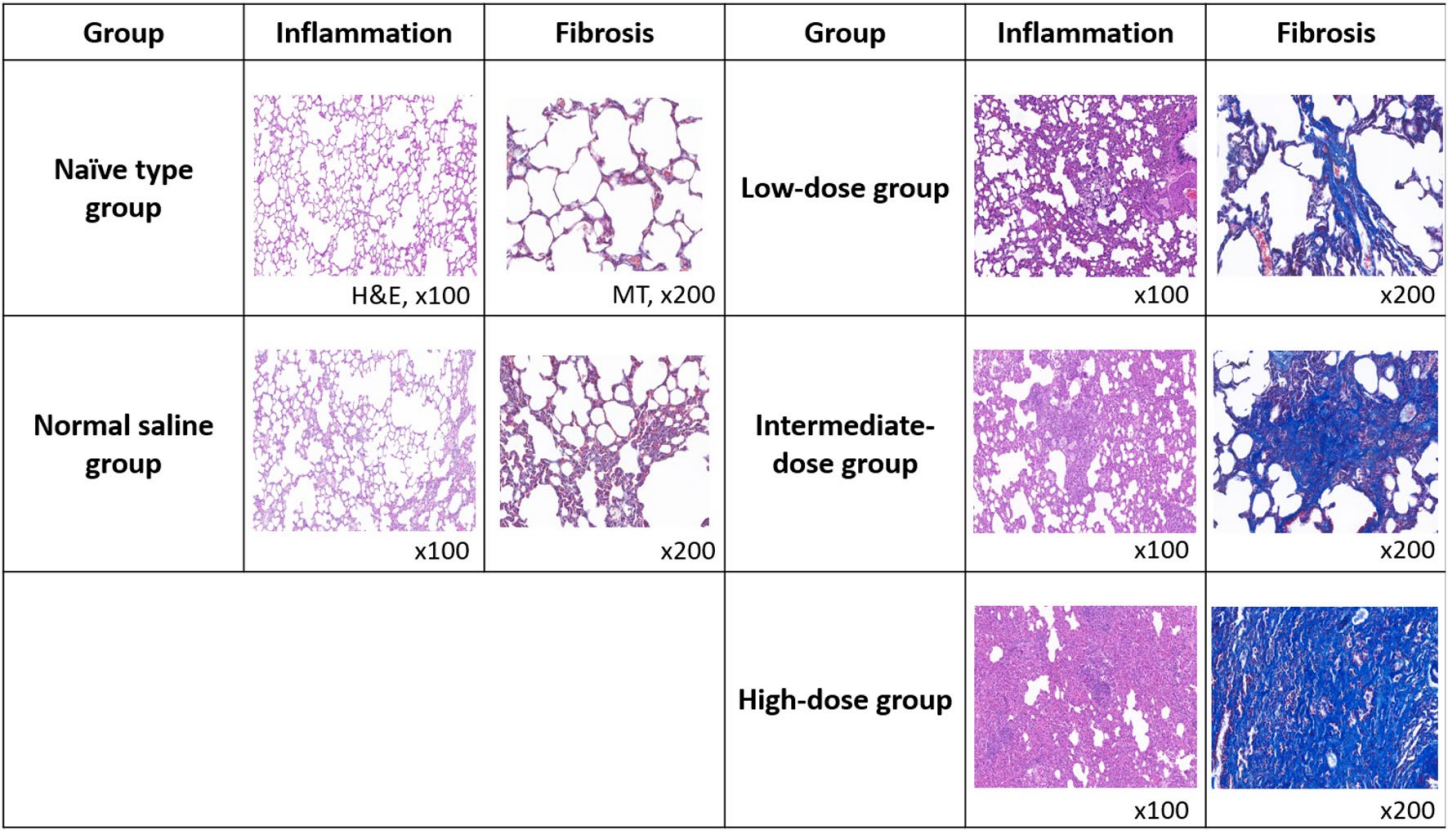
The summary of inflammation and fibrosis in all groups following histopathological analyses (hematoxylin and eosin stain was used to evaluate inflammation, and Masson’s trichrome stain was used to evaluate fibrosis).

Table 3 and Supplementary Fig. 2 show the intergroup comparisons of the histopathological changes from 10 to 40 weeks after the first intratracheal instillations. After 10 weeks, the mean inflammation and fibrosis scores tended to increase according to the PHMG-p dose. After 40 weeks, the mean inflammation and fibrosis scores were both significantly increased according to PHMG-p dose (inflammation score: naïve type group, 2.50 ± 0.76; normal saline group, 3.44 ± 0.88; low-dose group, 3.88 ± 0.35; intermediate-dose group, 3.67 ± 0.52; and high-dose group, 3.80 ± 0.45; P = 0.002; fibrosis score: naïve type group, 1.25 ± 0.71; normal saline group, 1.89 ± 0.33; low-dose group, 2.13 ± 0.53; intermediate-dose group, 3.83 ± 0.41; high-dose group, 4.80 ± 0.45; P < 0.001). The mean inflammation scores in the low-, intermediate-, and high-dose groups were significantly higher than in the naïve type group (all P < 0.05). The mean fibrosis scores in the intermediate- and high-dose groups were significantly higher than in the naïve type, normal-saline, and low-dose groups (all P < 0.05). Additionally, the mean fibrosis score in the high-dose group was significantly higher than in the intermediate-dose group (P < 0.05) (Fig. 2).

**Table 3:** The comparison of pathologic results for 10 weeks vs. 40 weeks after the first intratracheal instillation.

After 10 weeks				
Group	Inflammation score	Fibrosis score		
Naïve type	2.00 ± 0.00	0.50 ± 0.71		
Normal saline	3.00 ± 0.00	2.00 ± 0.00		
Low-dose	3.50 ± 2.12	1.50 ± 0.71		
Intermediate-dose	4.25 ± 0.96	4.00 ± 0.00		
High-dose	4.75 ± 1.26	4.50 ± 0.58		

After 40 weeks				
Group	Inflammation score	P-value	Fibrosis score	P-value
Naïve type	2.50 ± 0.76	0.002	1.25 ± 0.71	< 0.001
Normal saline	3.44 ± 0.88		1.89 ± 0.33	
Low-dose	3.88 ± 0.35*		2.13 ± 0.53	
Intermediate-dose	3.67 ± 0.52*		3.83 ± 0.41*^†‡^	
High-dose	3.80 ± 0.45*		4.80 ± 0.45*^†‡§^	

*P < 0.05 (vs. naïve type group), ^†^P < 0.05 (vs. normal saline group), ^‡^P < 0.05 (vs. low-dose group), ^§^P < 0.05 (vs. intermediate-dose group).

### Tumors after 10 and 40 weeks

**Figure 3:**
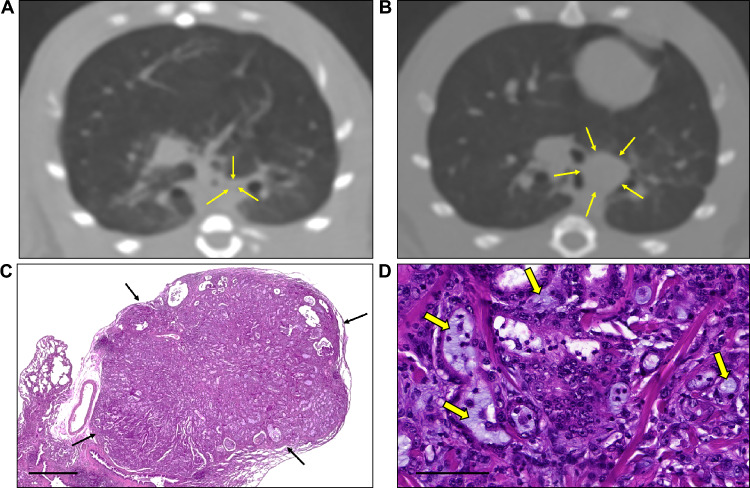
CT images and the histological findings for adenocarcinomas. (**A**) An axial chest CT image was taken 10 weeks after the first tracheal instillation; the left lobe had a 3.2 mm nodule (arrows). (**B**) Axial CT image after 40 weeks; the nodule size increased (5.3 mm, arrows). (**C**) Histopathological evaluation found this tumor (arrows) to be an adenocarcinoma. The invasive tumor was arranged in the acini and tubules and was composed of cuboidal or columnar cells that resembled bronchial glands or bronchial-lining epithelial cells (H&E, × 20; scale bar, 1mm). (**D**) The atypical glands were fused with mucin (arrows) (H&E, × 400; scale bar, 100μm).

**Figure 4:**
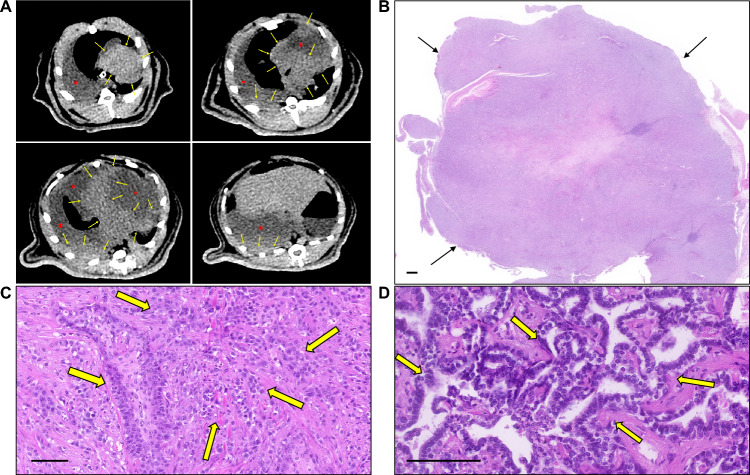
CT images and the histopathological findings of squamous cell carcinoma. (**A**) Axial chest CT images were taken 40 weeks after the first tracheal instillations; multiple tumors in both lungs (arrows) and malignant pleural effusion (red asterisks) are shown. (**B**) Histopathological evaluation found this tumor (arrows) to be a squamous cell carcinoma (H&E, ×6; scale bar, 1mm). (**C**) The tumor cells had cytologic features of malignancy (cellular atypia, disorganization, and increased mitotic rate (arrows)) (H&E, × 200; scale bar, 100μm). (**D**) The adenocarcinoma component (arrows) was observed in less than 5% of the tumor (H&E, × 400; scale bar, 100μm).

Table 4, Supplementary Table 1, and Supplementary Fig. 3 present the intergroup comparisons in the numbers of lung tumors. After 10 and 40 weeks, bronchiolar–alveolar hyperplasias had developed in the low-, intermediate-, and high-dose groups in a dose-dependent manner, with significantly increasing extent (P = 0.045 after 10 weeks and P < 0.001 after 40 weeks). After 10 weeks, bronchiolar–alveolar adenomas or epitheliomas were found in the intermediate- and high-dose groups. After 40 weeks, bronchiolar–alveolar adenomas and epitheliomas were found in the low- and high-dose groups, while carcinomas were found only in the high-dose group. Specifically, after 10 weeks, a total of two bronchiolar–alveolar hyperplasia lesions were observed in the low-dose group (in one of two rats), compared with 16 in the intermediate-dose group (in four of four rats) and 32 in the high-dose group (in four of five rats). After 40 weeks, 19 bronchiolar–alveolar hyperplasias (in six of eight rats) and one bronchiolar–alveolar adenoma (in one of eight rats) were found in the low-dose group; 40 bronchiolar–alveolar hyperplasias (in five of six rats) were found in the intermediate-dose group; 48 bronchiolar–alveolar hyperplasias (in five of five rats), four bronchiolar–alveolar adenomas/epitheliomas (in three of five rats), and 33 carcinomas (in two of five rats) were detected in the high-dose group. Among the 33 carcinomas in the two rats, one rat had an adenocarcinoma (Fig. 3), and the other had 32 squamous carcinomas. Among the 32 squamous carcinomas, the largest mass had an adenocarcinoma component within the tumor (Fig. 4). At 10 weeks, two tumors in this rat were identified, and by 40 weeks, each had increased in size (Supplementary Table 2). However, it cannot be conclusively asserted that all 30 lesions emerging after 10 weeks were metastatic. While the largest tumor exhibited an adenocarcinoma component, the lack of this component in the other lesions prevents a conclusive classification as metastatic. Hence, it is reasonable to consider the likelihood that these lesions primarily represent concurrent tumors.

**Table 4:** The comparison of pathologically proven lung tumors for 10 weeks vs. 40 weeks after the first intratracheal instillation.

Group	Bronchiolar–alveolar hyperplasia	P-value	Bronchiolar adenoma or epithelioma	P-value	Carcinoma	P-value
After 10 weeks						
Naïve type	0	0.045	0	0.850	0	N/A
Normal saline	0		0		0	
Low-dose (number of rats with lesion/total number of lesions)	1.00 ± 1.41 (1/2)		0		0	
Intermediate-dose (number of rats with lesion/total number of lesions)	4.00 ± 2.16 (4/16)		0.25 ± 0.50 (1/1)		0	
High-dose (number of rats with lesion/total number of lesions)	8.00 ± 4.55 (4/32)		0.25 ± 0.50 (1/1)		0	
After 40 weeks						
Naïve type	0	< 0.001	0	0.005	0	0.013
Normal saline	0		0		0	
Low-dose (number of rats with lesion/total number of lesions)	2.38 ± 1.77*^†^ (6/19)		0.13 ± 0.35 (1/1)		0	
Intermediate-dose (number of rats with lesion/total number of lesions)	6.67 ± 4.80* (6/40)		0		0	
High-dose (number of rats with lesion/total number of lesions)	7.80 ± 5.45*^†^ (5/48)		1.00 ± 1.00 (3/4)		6.60 ± 14.21 (2/33)	

*P < 0.05 (vs. naïve type group), ^†^P < 0.05 (vs. normal saline group).

### Longitudinal changes in lung tumors

Supplementary Table 2 and Supplementary Fig. 4 show the results of the lesion-to-lesion histopathological analyses, which confirmed lung tumors after 40 weeks. All tumors after 40 weeks were confirmed as bronchiolar–alveolar hyperplasias, bronchiolar–alveolar adenomas, epitheliomas, or carcinomas by CT. All bronchiolar–alveolar hyperplasias in the intermediate- and high-dose groups were detected by CT after 10 weeks, whereas 84.2% (16 of 19 rats) of the bronchiolar–alveolar hyperplasias were detected in the low-dose group. CT scans detected all bronchiolar adenomas, epitheliomas, and carcinomas after 10 weeks, and all these tumors increased in size on the follow-up CT scan after 40 weeks compared with the sizes at week 10 (Supplementary Figs. 5 and 6). The size of the bronchiolar–alveolar hyperplasias at week 40 had either grown, shrunk, or remained unchanged compared to those observed at week 10 (Supplementary Figs. 7 and 8). The mean size of the bronchiolar–alveolar hyperplasias found in the high-dose group was significantly higher at weeks 10 and 40 than those in the low- and intermediate-dose groups (P = 0.022 and P < 0.001). However, carcinoma was found only in the high-dose group after 40 weeks.

## Discussion

This study revealed longitudinal changes in lung injuries according to the PHMG-p dose using repeated chest CT scans in a rat model and a long-term follow-up. The lung lesions identified on the chest CT after 10 weeks persisted without significant changes even after 40 weeks. As the dose of PHMG-p increased, lung damage became more severe. The number of bronchiolar–alveolar hyperplasias significantly increased as the dose increased. The mean size of the bronchiolar–alveolar hyperplasias in the high-dose group was significantly higher after 10 and 40 weeks than those in the low- and intermediate-dose groups. However, carcinoma was found only in the high-dose group after 40 weeks.

The volume of the lung lesions was dependent on the PHMG-p dose, with the mean lesion volume percentage in the high-dose group after 10 and 40 weeks being significantly larger than those in the low- and intermediate-dose groups. In the histopathological analyses, the mean fibrosis scores were significantly increased as the dose increased at 10 and 40 weeks, and the mean fibrosis scores in the intermediate- and high-dose groups were significantly higher than those in the low-dose group. These trends were also observed in a previous epidemiological study in South Korea, whereby a dose–response analysis indicated a strong association between lung injury/death and recurrent and intense HD exposure without sufficient recovery time between exposures^19^. Since this previous epidemiological study using a short-term rat model had indicated that 2 weeks was insufficient for lung injuries to recover fully, our study involved intratracheal instillation of PHMG-p every 2 weeks to mimic the recurrent and intense exposures without sufficient recovery time between exposures^5^. Furthermore, a minimum duration of intense exposure is required for humans to develop HD-associated lung injuries^19^. Since the cumulative dose in the low-dose group in our study was 0.2 mg/kg, further research using a dose less than 0.2 mg/kg is needed to evaluate the minimum threshold for lung injury from PHMG-p exposure.

Lung lesions on the chest CT in our study were defined as those that involved both fibrosis and inflammation since distinguishing pure fibrosis from inflammation only using lung densities (HU) in the chest CT scan is not easy. To minimize analysis errors, we relied on a board-certified radiologist with 12 years of experience in thoracic imaging to determine the presence or absence of lung inflammation or fibrosis in all chest CT images. Our results showed no statistically significant differences between the respective PHMG-p-treated groups regarding lung lesion volumes between the 10- and 40-week observations, meaning that fibrosis and inflammation persisted 40 weeks after the PHMG-p exposure. In the histopathological analyses, the mean inflammation and fibrosis scores for each PHMG-p exposure group were similar between the 10- and 40-week observations. Although we did not directly compare the histopathological scores between the 10- and 40-week observations—because there were not enough rats after 10 weeks—these results might reflect the trends of persistent lung lesions even with low-dose exposure to PHMG-p after 40 weeks.

In our study, the number, size, and malignancy of lung tumors were associated with the PHMG-p dose. The number of bronchiolar–alveolar hyperplasias significantly increased as the dose increased. The mean size of the bronchiolar–alveolar hyperplasias in the high-dose group was significantly higher than those in the low- and intermediate-dose groups, both after 10 and 40 weeks. Carcinomas were found only in the high-dose group using 1.0 mg/kg of PHMG-p after 40 weeks. Similar results were also observed in a previous study using a single-exposure PHMG-p dose of 0.9 mg/kg, which significantly increased the number of bronchiolar–alveolar hyperplasia lesions at 52 weeks compared with 8 and 26 weeks; carcinoma development was also observed after exposure at 52 weeks^6^. That study also demonstrated several upregulated genes associated with lung cancer and tumor metastasis^6^. These results indicate that the number and malignancy of lung tumors are increased and exacerbated in a PHMG-p dose-dependent manner and that PHMG-p could be a possible lung carcinogen.

In the longitudinal chest CT follow-up of the present study, the size of the bronchiolar–alveolar hyperplasia lesions either grew, shrunk, or remained unchanged after 40 weeks compared with those observed at 10 weeks. In contrast, the bronchiolar adenomas, epitheliomas, and carcinomas increased in size after 40 weeks compared with those at 10 weeks. We could not confirm the histopathological features of the detected tumors on the CT scans after 10 weeks; however, these results suggest that size increases by the bronchiolar–alveolar hyperplasias varied. Conversely, the bronchiolar–alveolar adenomas and carcinomas usually increase in size over time. Therefore, the bronchiolar–alveolar hyperplasia lesions were pluripotent. Previously, the theory of the bronchiolar-alveolar hyperplasia→adenoma→carcinoma sequence has been raised in several studies^5,6,20^. Given our finding that a direct transition from bronchiolar–alveolar hyperplasia to carcinoma could be possible, further research on this topic is warranted.

There were some limitations in this study. First, no study has previously investigated the precise molecular mechanism underlying PHMG-induced lung carcinogenesis. As the primary goal of this study was assessed by histopathological and CT analyses after PHMG-p instillation, future research should focus on the molecular mechanisms involved. Second, as previously mentioned, we did not directly compare the histopathological scores between the 10- and 40-week lungs because there were not enough rats after 10 weeks. However, even though we had originally planned to evaluate longitudinal changes using chest CT scans, we still conducted histopathological evaluations in the rats that unexpectedly died after 10 weeks. Third, we did not assess tumors detected by CT after 10 weeks but had disappeared on the CT at 40 weeks. Therefore, we cannot conclude from this study regarding the possibility of spontaneous resolution of lung tumors in the investigated follow-up period. Fourth, we assessed only the tumor size regardless of the lung nodule characteristics, such as solid or subsolid patterns. As atypical adenomatous hyperplasia in humans is usually seen as subsolid nodules (which have malignant potential), changes from subsolid nodules to solid nodules upon follow-up are important for predicting prognosis. Future research should be designed to evaluate changes in nodule characteristics. Lastly, intratracheal liquid instillation has limitations and potential drawbacks in animal models. However, this technique is a common method used in research to deliver substances directly into the lungs. One of the significant limitations is the potential for inhomogeneous distribution of the instilled liquid within the lungs. Moreover, the dose administered intratracheally cannot be converted to an inhaled concentration. The procedure often requires anesthesia, which can affect respiratory function and may introduce additional variables into the experimental design.

In conclusion, this study found that lung damage in a rat model due to PHMG-p was dose-dependent using repeated chest CT scans with a long-term follow-up. The lung lesions, including lung fibrosis and inflammation, seen on the chest CT scans after 10 weeks persisted without significant changes, even at 40 weeks. The numbers and malignancy of the lung tumors were also dose-dependent, meaning PHMG-p could be a possible lung carcinogen in rats.

## Materials and methods

This study was approved by the Institutional Animal Care and Use Committee of the Korea University Medical Center (approval number: KOREA-2021–0051-C1). This study complied with the ARRIVE (Animal Research: Reporting of In Vivo Experiments) guidelines and all experiments were performed per Korea University guidelines.

### Animals

Seven-week-old male Sprague–Dawley rats (Raonbio, Yong-in, South Korea) were acclimated for 1 week (three rats per cage). The conditions were as follows: temperature: 22–25 ℃; relative humidity: 40–60%; lighting conditions: 12-h light/dark cycles. Experimental rodents (Purina, Sung-nam, South Korea) were provided pelleted food and filtered tap water ad libitum.

### Experimental design

The experimental design is summarized in Supplementary Fig. 9. A total of 50 rats were randomly divided into five groups: naïve type group (10 rats), normal saline group (10 rats), low-dose group (10 rats), intermediate-dose group (10 rats), and high-dose group (10 rats). All rats were anesthetized using 2% isofluorane in 70% N_2_O and 30% O_2_ for intratracheal instillation of low-, intermediate-, high-dose PHMG-p, and normal saline. The determination of the PHMG-p exposure dose was predicated upon examining concentrations administered in antecedent studies and the actual exposure levels of humans in Korea. Upon conversion of the exposure concentrations employed in the inhalation toxicity investigation conducted by the Korea Centers for Disease Control and Prevention^21^ and another previous study^8^ into intratracheal instillation doses, the corresponding values were 2.08 mg/kg and 1.85 mg/kg, respectively. Moreover, the dosage employed in the report of the intratracheal instillation study by the National Institute of Environmental Research amounted to 2.0 mg/kg^22^. Furthermore, in our prior study, administering a PHMG-p dose of "0.9 mg/kg" through tracheal instillation in a rat model resulted in lung carcinomas occurring at 52 weeks^6^. Therefore, the respective groups (except the naïve type group) were treated with five doses of normal saline (0.05 mL/kg), low-dose (0.04 mg/kg), intermediate-dose (0.2 mg/kg), and high-dose (1.0 mg/kg) of PHMG-p every 2 weeks: the total amounts of PHMG-p were 0.2 mg/kg in the low-dose group, 1.0 mg/kg in the intermediate-dose group, and 5.0 mg/kg in the high-dose group.

CT examinations were performed under anesthesia on all rats using intraperitoneal and intramuscular injections of alfaxalone (30 mg/kg) and xylazine (10 mg/kg), respectively, 10 weeks after the first intratracheal instillation. CT scans were taken after anesthesia and tracheal intubation through the mouth of the rat. Some rats died after undergoing CT; this occurred more frequently among rats with compromised lungs: two in the naïve type group, one in the normal saline group, two in the low-dose group, four in the intermediate-dose group, and five in the high-dose group. Both lungs of each rat that died after CT examination were additionally evaluated histopathologically. Forty weeks after the first treatment (30 weeks after the first CT scan), the CT scan was repeated for all rats except those that had died. Subsequently, the animals were sacrificed, and both lungs were collected for histopathological evaluation.

### CT protocol

All CT images were captured using a Philips IQon 128-slice dual-layer detector spectral CT scanner (Philips Healthcare, Cleveland, OH, USA) with the subject in the supine position. All images were obtained in a caudocranial direction from the lung base through the thoracic inlet level during full inspiration breath-hold using a ventilator for small animals (VentElite, Harvard Apparatus, MA, USA). The scan time for one rat was less than 10 s. The CT scan parameters were as follows: 80 kVp; 400 mA; collimation: 64 × 0.625 mm; slice thickness: 0.67 mm; beam width: 40 mm; pitch: 1.048; rotation time: 0.4 s.

### CT evaluation

All CT images were quantitatively analyzed regarding the distribution of HU of the pixels by computing normalized histograms using commercial software (IntelliSpace Portal, Philips). As HU values of fibrotic lung lesions are typically within − 200 and − 600 HU, according to previous studies^23–25^, the definition of a lung lesion in the rat lung CT images was set as the area with HU values between − 200 and − 600 HU. Additionally, some lesions were identified outside of this definition if the examining radiologist regarded them to be lung lesions (including inflammation and fibrosis). The lesion volume (volume of lung lesion (mL)), whole-lung volume, and lesion volume percentage (volume of lung lesion/whole-lung volume × 100, lesion volume (%)) were measured and calculated. Additionally, all CT image pairs from 10 and 40 weeks after the first treatment were evaluated and compared. All CT lesions identified after 10 weeks were followed and compared with those after 40 weeks. One board-certified radiologist with 12 years of experience in thoracic imaging (C.K.) performed all procedures, CT image readings, and analyses while being blinded to the groups.

### Histologic examination

All extracted lung specimens were evaluated by one experienced pathologist with 22 years of experience in thoracic pathology (J.L.) who was blinded to the groups. The lungs were fixed in 10% neutral buffered formalin. Paraffin sections were cut into 4 µm from the fixed samples before hematoxylin and eosin (H&E) staining, and Masson’s trichome (MT) staining were performed.

Based on the extent (none, lesions involving 0% to 25%, > 25% to 50%, or > 50% of the total lung area) and severity (none, mild, moderate, or severe) of inflammation, the inflammation scores were calculated by adding the extent and severity of inflammation, with extent scored as 0 = none, 1 = lesions involving 0% to 25% of the lung, 2 = lesions involving > 25% to 50% of the lung, and 3 = lesions involving > 50% of the lung). Severity was scored as 0 = none, 1 = mild, 2 = moderate, and 3 = severe. A modified Ashcroft score was used to quantify fibrosis^26^. Additionally, the numbers of bronchiolar–alveolar hyperplasia lesions, bronchiolar–alveolar adenomas, epitheliomas, and carcinomas were evaluated in each group.

All pathologically confirmed tumors after 40 weeks were correlated with the CT findings by a radiologist (C.K.), and all lung tumors found on the CT images after 40 weeks were evaluated for their previous presence or absence on the CT images from 10 weeks after the study treatments (lesion-to-lesion comparison). The numbers of tumors detected after 10 weeks from the first intratracheal instillation and the sizes of the tumors, both after 10 and 40 weeks, were evaluated.

### Statistical analysis

Paired t-tests compared the quantitative CT analysis results between 10 and 40 weeks after the first intratracheal instillations. The Kruskal–Wallis rank-sum test and/or analysis of variance were conducted for intergroup comparisons of 10- and 40-week post-treatment CT and histopathological findings. The Kruskal–Wallis rank-sum test was also conducted to compare the numbers of lung tumors between the groups. Post hoc, Bonferroni correction for multiple comparisons was performed. All statistical analyses were performed using R, version 4.2.1 (R Foundation for Statistical Computing, Vienna, Austria). All P-values < 0.05 were considered statistically significant.

### Supplementary Information

Supplementary Information.

## Data Availability

All data generated or analyzed during this study are included in this published article.

## Abbreviations

PHMG-p: Polyhexamethylene guanidine phosphate
HD: Humidifier disinfectant
CT: Computed tomography
PHMB: Polyhexamethylene biguanide
