# Supplementary material for: Longitudinal long term follow up investigation on the carcinogenic impact of polyhexamethylene guanidine phosphate in rat models
Source: Sci Rep. 2024 Mar 26;14:7178. doi: 10.1038/s41598-024-57605-x (PMC10965910; doi:10.1038/s41598-024-57605-x)
Supplement: Supplementary file 1 — Supplementary Information. [file 41598_2024_57605_MOESM1_ESM.docx]

Supplementary Fig. 1. Graphs showing the comparisons of mean lesion volume (mL), mean lesion volume percentage, and mean whole lung volume (mL) from quantitative analysis of CT results from (A) 10 weeks and (B) 40 weeks after the first intratracheal instillations in each study group.

(A)


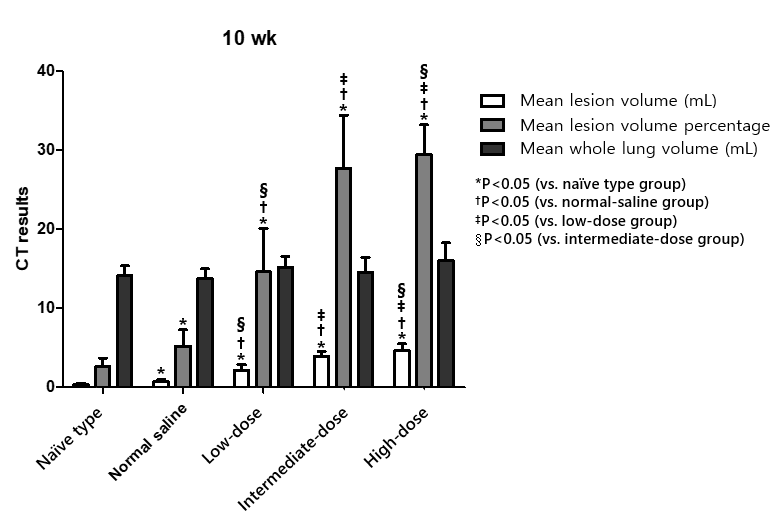


(B)


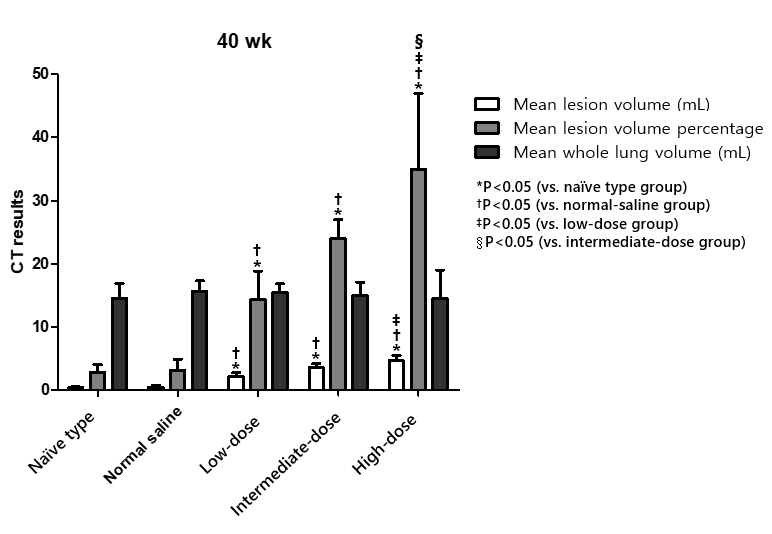


Supplementary Fig. 2. Graphs showing the comparisons of inflammation score and fibrosis score from (A) 10 weeks and (B) 40 weeks.

(A)


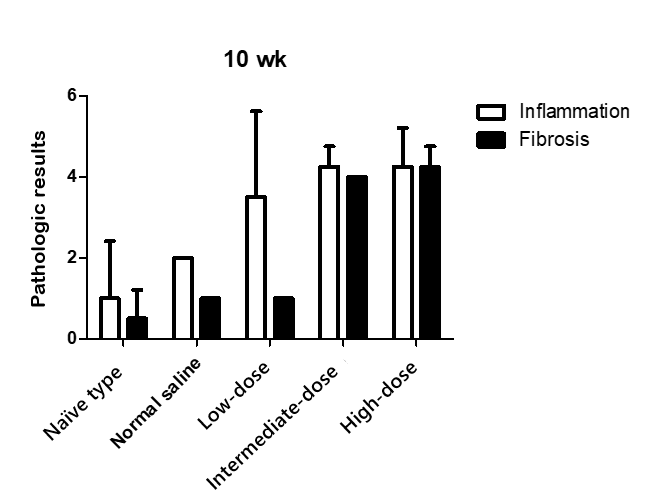


(B)


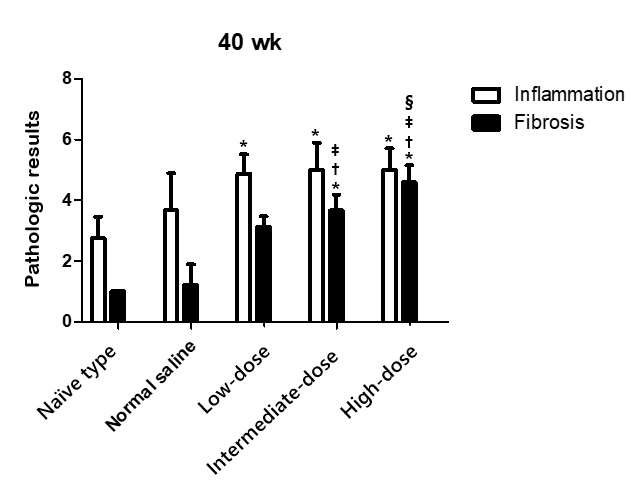


Supplementary Fig. 3. Graph showing the intergroup comparisons of the numbers of lung tumors.


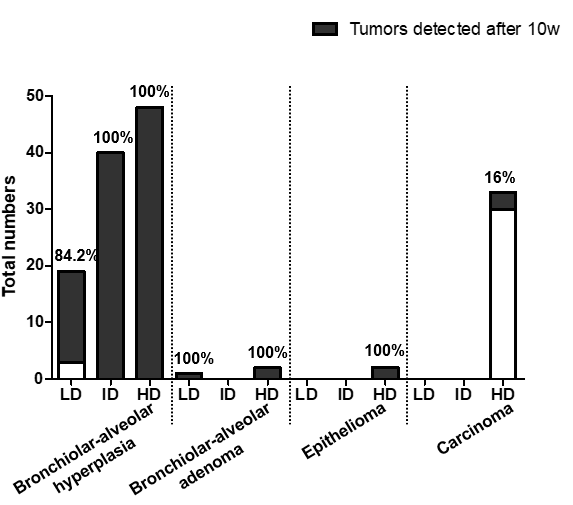


Supplementary Fig. 4. Graph showing the results of the lesion-to-lesion analyses of all histopathologically confirmed lung tumors after 40 weeks. The bronchiolar-alveolar hyperplasia after 40 weeks either grew, shrunk, or remained unchanged in terms of size compared with the 10-week findings.


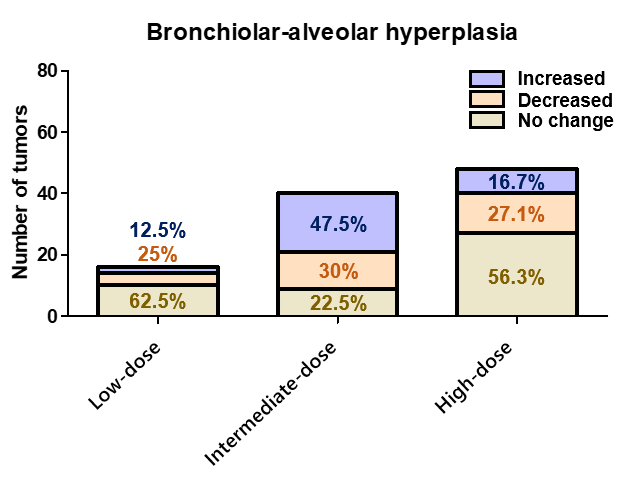


Supplementary Fig. 5. CT images and the histopathologic findings of bronchiolar-alveolar adenoma. (A) Axial chest CT image taken after 10 weeks from the first tracheal instillations; there was a 0.3-mm nodule in the left lobe (arrows). (B) Axial CT image after 40 weeks; the nodule size increased (2.6 mm, arrows). (C) Histopathological evaluation found this tumor to be a bronchiolar–alveolar adenoma. The lesion was well-circumscribed and had a sharp demarcation from the surrounding normal alveoli (H&E, ×60). (D) The tumor had a high epithelial cell density. The neoplastic epithelial cells were uniform. The mitotic figure was absent (H&E, ×200).


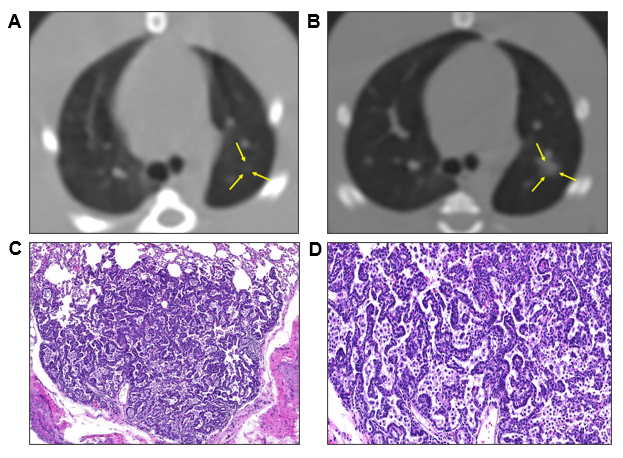


Supplementary Fig. 6. CT images and the histopathologic findings of epithelioma. (A) Axial chest CT image taken after 10 weeks from the first tracheal instillations; there was a 1.2-mm nodule in the right posterior lobe (arrows). (B) Axial CT image after 40 weeks; the nodule size increased (2.1 mm, arrows). (C) Histopathological evaluation found this tumor to be an epithelioma. Under low magnification, this tumor was cystic and clearly distinguished from the surrounding normal alveoli (H&E, ×100). (D) Microscopically, the multilayered wall of the tumor consisted of squamous cells with a central area of keratin (H&E, ×200, arrows).


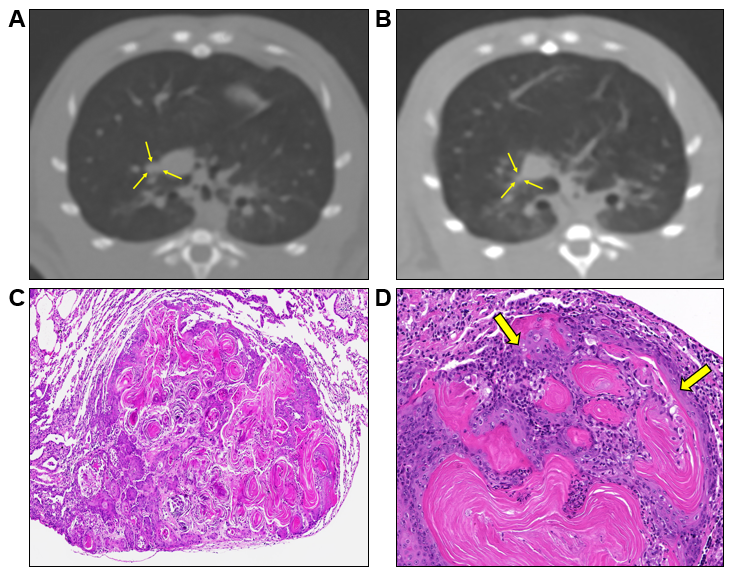


Supplementary Fig. 7. CT images and the histopathologic findings of bronchiolar-alveolar hyperplasia. (A) Axial chest CT image taken after 10 weeks from the first tracheal instillations; there was a 1.3-mm ground-glass nodule in the right superior lobe (arrows). (B) Axial CT image after 40 weeks; the nodule size decreased and density increased (0.9 mm, arrows). (C) On histopathologic evaluation, this lesion was proven to be bronchiolar-alveolar hyperplasia. Alveolar walls were lined by cuboidal to tall columnar cells (arrows) (H&E, ×200).


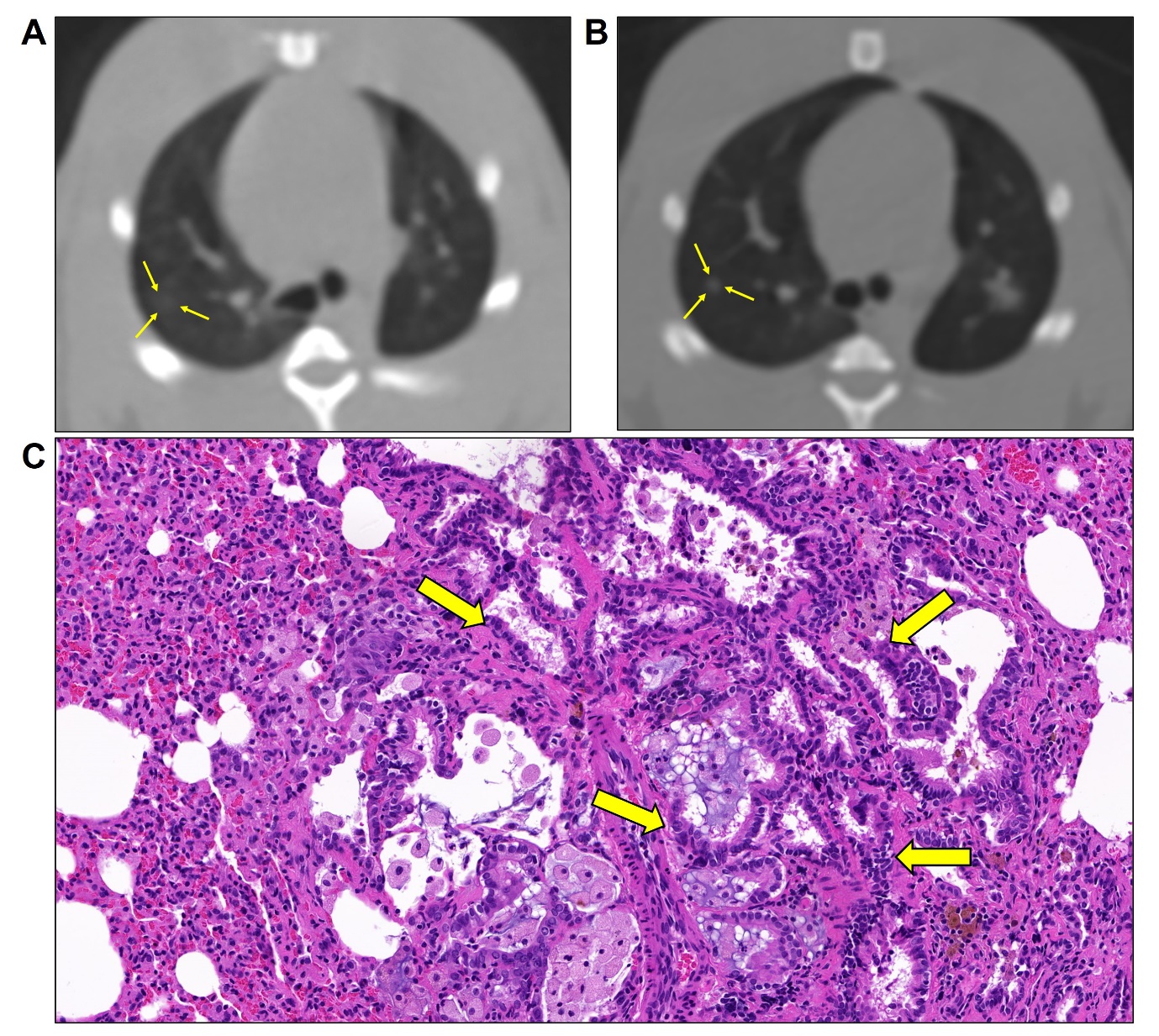


Supplementary Fig. 8. CT images and the histopathologic findings of bronchiolar-alveolar hyperplasia. (A) Axial chest CT image taken after 10 weeks from the first tracheal instillations; there was a 0.3-mm ground-glass nodule in the right posterior lobe (arrows). (B) Axial CT image after 40 weeks; the nodule size increased (0.5 mm, arrows). (C) On histopathologic evaluation, this lesion was proven to be bronchiolar-alveolar hyperplasia. The lesion shows indistinct borders. Alveolar walls were lined by cuboidal to tall columnar cells (arrows) (H&E, ×200).


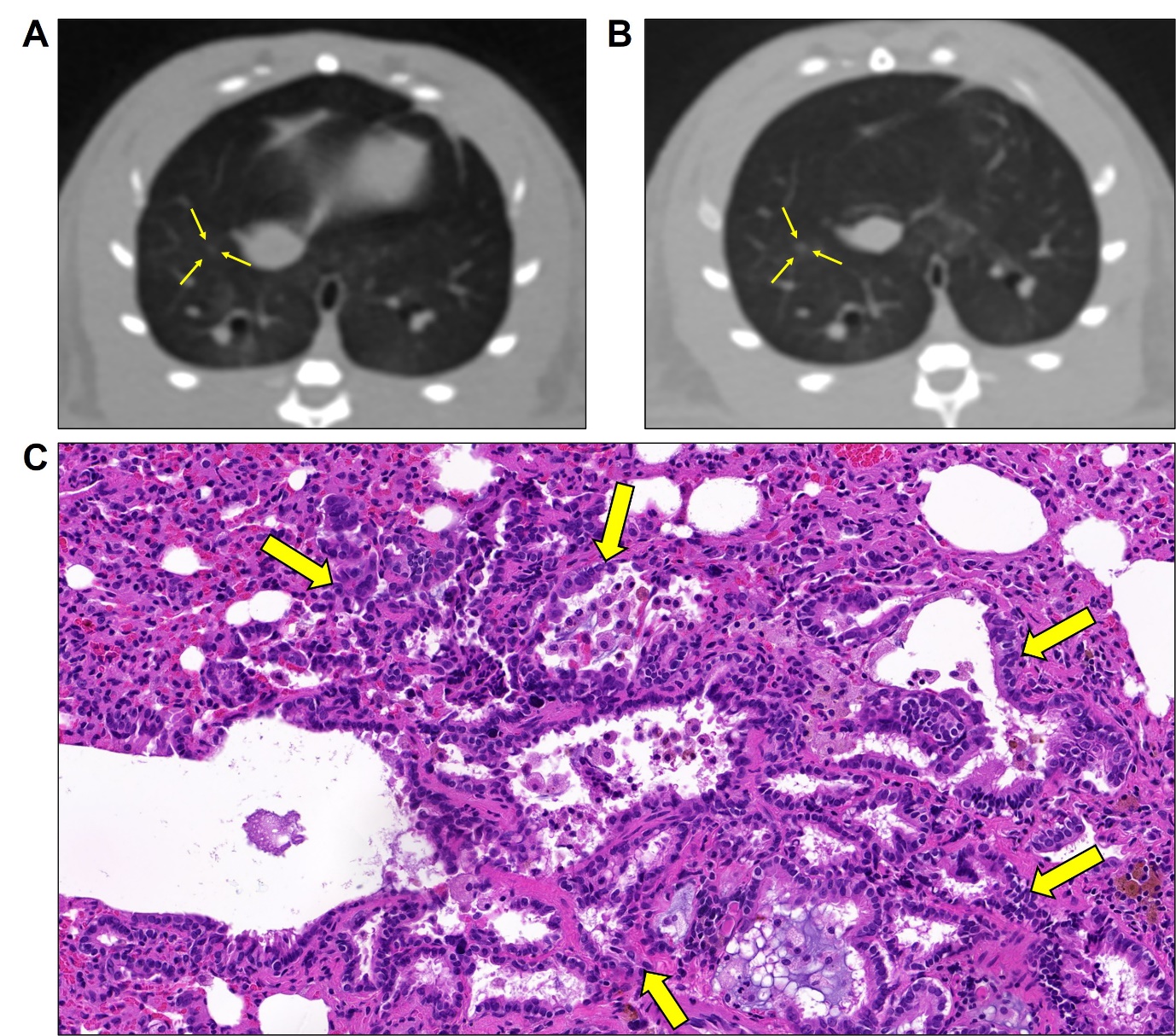


Supplementary Fig. 9. The summary of the experimental design.


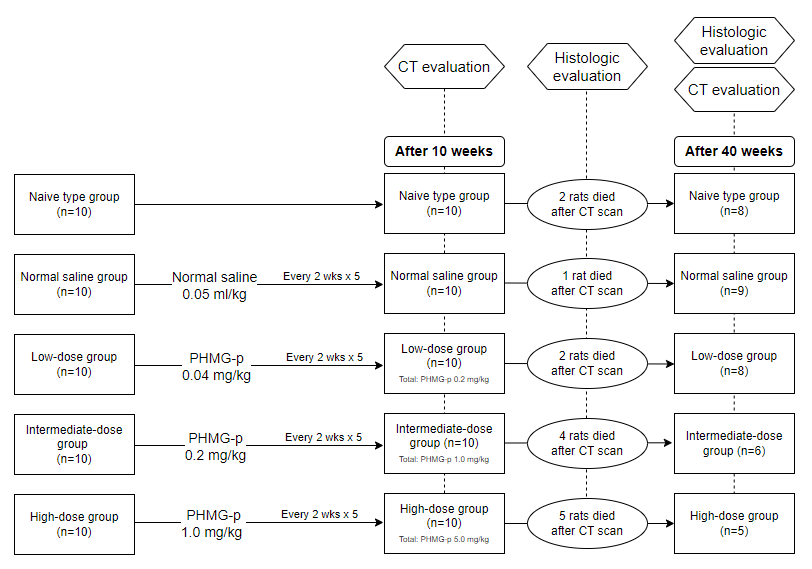


|  | **No tumor present** | | **Bronchiolar-alveolar hyperplasia** | | **Bronchiolar-alveolar adenoma/Epihelioma** | | **Carcinoma** | |
| --- | --- | --- | --- | --- | --- | --- | --- | --- |
|  | **10 weeks** | **40 weeks** | **10 weeks** | **40 weeks** | **10 weeks** | **40 weeks** | **10 weeks** | **40 weeks** |
| **Low-dose group** | N/A | 0 | 1 of 2 rats | 6 of 8 rats | N/A | 1 of 8 rats | N/A | 0 |
| **Intermediate- dose group** | N/A | 0 | 4 of 4 rats | 5 of 6 rats | N/A | 0 | N/A | 0 |
| **High-dose group** | N/A | 0 | 4 of 5 rats | 5 of 5 rats | N/A | 3 of 5 rats | N/A | 2 of 5 rats |

Supplementary Table 1. Number of rats at each group which presented neoplastic and non-neoplastic lesions at 10 and 40 weeks.

Note—N/A, not applicable.

Supplementary Table 2. The results of lesion-to-lesion analysis of all pathologically proven lung tumors after 40 weeks from the first intratracheal instillation.

| **Pathology** | **Group** | **Total number of tumors** | **Number of tumors detected after 10 weeks** | **Mean size after 10 weeks (mm)** | | **Mean size after 40 weeks (mm)** | | **Size change** |
| --- | --- | --- | --- | --- | --- | --- | --- | --- |
| **Bronchiolar-alveolar hyperplasia**  **(n=107)** | Low-dose | 19 | 16 (84.2%) | 0.68±0.36 | P=0.022 | 0.72±0.22 | P<0.001 | Increased: 10 (62.5%) Decreased: 4 (25%) No change: 2 (12.5%) |
|  | Intermediate-dose | 40 | 40 (100%) | 0.78±0.31 |  | 0.73±0.21 |  | Increased: 9 (22.5%) Decreased: 12 (30%) No change: 19 (47.5%) |
|  | High-dose | 48 | 48 (100%) | 1.23±1.43*† |  | 1.27±0.79*† |  | Increased: 27 (56.3%) Decreased: 13 (27.1%) No change: 8 (16.7%) |
| **Bronchiolar-alveolar adenoma**  **(n=3)** | Low-dose | 1 | 1 (100%) | 0.7 | | 0.8 | | Increased: 1 (100%) |
|  | Intermediate-dose | 0 | 0 | N/A | | N/A | | N/A |
|  | High-dose | 2 | 2 (100%) | 0.25±0.71 | | 2.15±0.63 | | Increased: 2 (100%) |
| **Epithelioma**  **(n=2)** | Low-dose | 0 | 0 | N/A | | N/A | | N/A |
|  | Intermediate-dose | 0 | 0 | N/A | | N/A | | N/A |
|  | High-dose | 2 | 2 (100%) | 1.05±0.07 | | 2.45±0.64 | | Increased: 2 (100%) |
| **Carcinoma**  **(n=33)** | Low-dose | 0 | 0 | N/A | | N/A | | N/A |
|  | Intermediate-dose | 0 | 0 | N/A | | N/A | | N/A |
|  | High-dose | 33 | 3 | 6.73±3.10 | | 17.77±12.35 | | Increased: 3 (100%) |
| *P<0.05 (vs. low-dose group) †P<0.05 (vs. intermediate-dose group)  N/A, not applicable. | | | | | | | | |
